# How to Be a Man

**Published:** 1878-07

**Authors:** 


How to be a Man.—When Carlysle was asked by a young person to point out what course of reading he thought best to make him a man, replied, in his characteristic manner. The letter is too long—we quote only the concluding paragraph :—
" In conclusion, I will remind you that it is not books alone, or by books chiefly, does a man become in all points a man. Study to do faithfully whatsoever thing in your actual situation, then and how, you find either expressly or tacitly laid down to your charge—that is your post—stand in it like a true soldier. Silently devour the many chagrins of it, as all situations have many, and see you aim not to quit it without doing all that it at least required of you. A man perfects himself by work much more than by reading. There are a growing kind of men that can wisely combine the two things—wisely, valiantly, can do what is laid to their hand in the present sphere, and prepare themselves withal for doing other wider things, if such lie before them."—*Carlisle.*

